# Association between health literacy and kinesiophobia in patients after percutaneous coronary intervention

**DOI:** 10.3389/fpsyg.2026.1689455

**Published:** 2026-07-02

**Authors:** Lijuan Lin, Haiyan Xia, Limin Yang

**Affiliations:** Department of Cardiovascular Medicine I, Meizhou People's Hospital, Meizhou, China

**Keywords:** coronary heart disease, health literacy, interaction effect, kinesiophobia, percutaneous coronary intervention (PCI)

## Abstract

**Objective:**

Kinesiophobia is a common barrier in the postoperative rehabilitation process of cardiovascular disease patients. This study aimed to investigate the association between health literacy and kinesiophobia in patients after Percutaneous Coronary Intervention (PCI).

**Methods:**

This cross-sectional study included 168 patients who had undergone PCI. Based on their scores on the Tampa Scale for Kinesiophobia-Heart (TSK-Heart), patients were divided into high and low kinesiophobia groups. Univariate analyses were performed to compare baseline characteristics between the two groups. Three multivariate logistic regression models were constructed to analyze the association between health literacy and kinesiophobia after adjusting for different covariates. Restricted cubic spline (RCS) models were used to explore the potential nonlinear relationship between health literacy and kinesiophobia. Receiver operating characteristic (ROC) curve analysis was conducted to evaluate the discriminative ability of health literacy in identifying high versus low kinesiophobia. Interaction and stratified analyses were also performed to assess the effect modification of other variables on this association.

**Results:**

Patients in the high kinesiophobia group had lower education levels, longer disease duration, poorer cardiac function, and higher rates of previous myocardial infarction and multiple stent implantations (*p* < 0.05). In all multivariate logistic regression models, patients with higher health literacy levels (HeLMSQ3 and HeLMSQ4) had significantly lower odds of high kinesiophobia (*p* < 0.05). RCS analysis showed a significant linear or near-linear negative association between health literacy and kinesiophobia. Interaction analysis indicated that education level and NYHA cardiac function classification significantly modified the association, with the protective effect of health literacy being more prominent in patients with higher education and better cardiac function.

**Conclusion:**

The results of this study indicate that health literacy is negatively associated with kinesiophobia in patients after PCI, and a linear or near-linear relationship may exist between the two. This association is more pronounced in patients with higher education levels and better cardiac function.

## 1 Introduction

Coronary heart disease (CHD), also referred to as coronary artery disease (CAD), is a condition in which atherosclerotic changes in the coronary arteries lead to stenosis or occlusion, resulting in myocardial ischemia, hypoxia, or necrosis. In China, the number of people suffering from CHD is as high as 11.39 million, and the mortality rate is increasing year by year, placing enormous pressure on medical resources and the public health system (Dalen et al., 2014; Mi et al., 2023). Percutaneous coronary intervention (PCI) has become one of the main treatments for CHD, primarily aimed at relieving vascular stenosis and improving myocardial ischemia (Hoole and Bambrough, 2020; Kandan and Johnson, 2019). Although PCI has the advantages of minimal trauma, significant therapeutic effects, and high safety, it may still face challenges such as restenosis and myocardial infarction post-procedure. Therefore, cardiac rehabilitation after PCI is particularly important to promote recovery of cardiac function, enhance self-management ability, and improve health literacy.

Health literacy refers to a patient’s ability to understand health-related information and use it to make informed decisions (Hersh et al., 2015; van der Gaag et al., 2022). Kinesiophobia, or fear of movement, refers to an irrational fear of exercise due to concerns that physical activity may negatively impact the disease (Luque-Suarez et al., 2019). Exercise is essential for the postoperative recovery of PCI patients, as appropriate physical activity can improve cardiopulmonary function, promote blood circulation, control weight, and regulate blood glucose and lipid levels (Dibben et al., 2023). However, kinesiophobia often leads patients to avoid exercise, potentially affecting their quality of life and long-term survival. Previous studies have shown that kinesiophobia is a significant risk factor for adverse cardiovascular events in patients with acute myocardial infarction (Bäck et al., 2020), and health literacy has been found to be associated with such events (Rymer et al., 2018; Magnani et al., 2018).

Previous studies have analyzed kinesiophobia and its influencing factors in patients with coronary artery disease (Wang et al., 2023; Liu et al., 2024); however, no research has yet clarified the relationship between health literacy and kinesiophobia after PCI. This study aims to fill this gap by revealing the independent impact of health literacy on kinesiophobia and its potential mechanisms, providing a theoretical basis and practical guidance for postoperative rehabilitation interventions.

## 2 Materials and methods

### 2.1 Study subjects

Convenience sampling was used to select patients who underwent PCI surgery and were hospitalized in our department from January 2024 to August 2024 as the study subjects. Inclusion criteria: ① underwent PCI treatment in our department; ② age >18 years; ③ able to communicate normally; ④ possess certain reading ability. Exclusion criteria: ① combined with important organ dysfunction such as liver or kidney failure; ② patients with comorbid psychiatric disorders or cognitive impairments who are unable to complete the questionnaire; ③ combined with respiratory failure. Based on the principle that the sample size should be 5 to 10 times the number of variables, and considering the 17 variables included in this study, with an estimated sample loss of approximately 5%, the required sample size was calculated to be between 90 and 180 cases. Initially, 185 patients were assessed for eligibility, and after applying the inclusion and exclusion criteria, 168 patients were finally included in the study, corresponding to an inclusion rate of 90.8% (Supplementary Figure 1). All patients were fully informed of the study content and signed informed consent. The Ethics Committee of our hospital approved this study (Ethical approval number: Meishi Lunshen 2024-C-29).

### 2.2 Research methods

Baseline information of patients was collected through a general data questionnaire, including age, sex, body mass index (BMI), education level, marital status, monthly household income per capita, payment method, living arrangement, duration of disease, number of implanted stents, smoking and drinking status, history of angina pectoris, history of myocardial infarction, number of PCI surgeries, preoperative left ventricular ejection fraction (LVEF), and preoperative New York Heart Association (NYHA) cardiac function classification.

Patients’ health literacy levels were assessed using the Health Literacy Management Scale (HeLMS) for chronic disease patients. This scale is based on the Health Literacy Management Scale developed by Professor Jordan et al. in Australia. It mainly includes four dimensions: information acquisition ability, communication and interaction ability, willingness of economic support, and willingness to improve health. The scale consists of 24 items, each scored on a 1–5 scale, with a total score ranging from 24 to 120. Higher scores indicate higher levels of patient health literacy. The overall Cronbach’s *α* of this scale was 0.894, and the test–retest reliability was 0.683, indicating good internal consistency and moderate acceptable temporal stability.

Post-PCI kinesiophobia was evaluated by the Chinese version of the Tampa Scale for Kinesiophobia Heart (TSK-Heart), which was developed by Meng et al. (2024). This scale mainly includes four dimensions: perception of danger, exercise avoidance, fear of exercise, and functional disorder. It contains 17 items, each scored from 1 to 4 points, with a total score ranging from 17 to 68. According to commonly used classification criteria in the literature, patients scoring above 37 were classified as the high kinesiophobia group, while the others were classified as the low kinesiophobia group (Dąbek et al., 2020). Higher scores indicate higher levels of postoperative kinesiophobia in PCI patients. The overall Cronbach’s *α* coefficient of this scale was 0.859, and the test–retest reliability was 0.792, indicating relatively high internal consistency and temporal stability.

All patients completed the questionnaires and scales anonymously at the time of discharge. Researchers explained the purpose and significance of the study to the patients, and patients filled in the general data questionnaire, HeLMS, and TSK-Heart scale after surgery. Researchers collected the questionnaires and scales on site and checked the completeness. If there were any omissions, patients were asked to complete the missing parts promptly. A total of 170 questionnaires and scales were distributed in this study, with 168 actually collected, yielding an effective recovery rate of 98.82%.

### 2.3 Statistical analysis

All analyses and plotting in this study were performed using R software version 4.4.1. Continuous data were expressed as median (minimum–maximum) and analyzed using independent samples *t*-test or Mann–Whitney U test. Categorical data were expressed as frequency (percentage) and analyzed using chi-square test or Fisher’s exact test. According to patients’ total HeLMS scores, health literacy was grouped by quartiles, sequentially divided into HeLMS Q1 (lowest quartile), HeLMS Q2, HeLMS Q3, and HeLMS Q4 (highest quartile) groups. Three multivariate logistic regression models were constructed, with the dependent variable being high kinesiophobia versus low kinesiophobia. Model 1 included only health literacy grouping without adjusting for any confounders; Model 2 adjusted for confounders of age, sex, and BMI; Model 3 adjusted for other significant factors in univariate analysis except age, sex, and BMI, including education level, disease duration, number of implanted stents, number of PCI surgeries, preoperative LVEF level, and preoperative NYHA classification. Bootstrap resampling (1,000 iterations) was used for internal validation of the models, and calibration curves were constructed to evaluate model calibration performance. Using the “rms” package, a restricted cubic spline regression model (RCS) was applied, with the total HeLMS score as the independent variable and kinesiophobia grouping as the dependent variable, setting 3 knots to plot a smooth curve, exploring its nonlinear trend, and calculating the *p* value to determine whether linear and nonlinear components were significant. Receiver operating characteristic (ROC) curve analysis was used to evaluate the discriminative ability of health literacy for high versus low kinesiophobia. To facilitate interaction analysis, significant risk factors in this study were uniformly recoded in the direction of protective factors. Specifically, age and disease duration were dichotomized based on the median and reverse coded: values greater than or equal to the median were coded as 0, and those below the median as 1. Similarly, variables such as the number of stents, history of myocardial infarction, number of PCI procedures, and NYHA classification were also reverse coded, with more severe conditions coded as 0 and less severe or absent conditions coded as 1. The interaction model consisted of three components: the HeLMS group, the effect modifier (e.g., age and education level), and their interaction term. The model was analyzed using multivariable logistic regression. An OR greater than 1 for the interaction term indicates that the effect modifier may strengthen the association between HeLMS and kinesiophobia, whereas an OR less than 1 suggests that the effect modifier may attenuate this association. An interaction term *p* value <0.05 was considered indicative of a significant interaction. In addition, subgroup difference analyses were conducted to assess the strength of the relationship between health literacy and kinesiophobia in different populations. Multiple imputation based on chained equations was used to handle missing data, and multiple imputed datasets were generated and pooled for subsequent analyses.

## 3 Results

### 3.1 Differences in baseline characteristics between high and low kinesiophobia groups

The results showed that the median age of all patients was 61 years (range: 45–78 years). Males accounted for 65.48% and females for 34.52%. The median body mass index (BMI) was 24.3 kg/m^2^ (range: 19.7–28.4). Patients with a junior high school education or below accounted for 30.95%, those with a senior high school education for 36.31%, and those with a college diploma or above for 32.74%. Married individuals made up 72.02%, while others accounted for 27.98%. The proportion of patients with a per capita monthly household income below 5,000 RMB was 47.62, and 52.38% had an income of 5,000 RMB or above. Patients covered by medical insurance comprised 91.07%, and self-paying patients made up 8.93%. A total of 41.67% lived with children. The median duration of illness was 3.0 years (range: 0.3–6.4 years). Patients with fewer than 2 stents accounted for 52.98%, while those with 2 or more accounted for 47.02%. The proportion of patients with a history of smoking was 26.19%; with a history of alcohol consumption, 1.79%; with a history of angina, 43.45%; and with a history of myocardial infarction, 2.38%. Patients who underwent a single PCI procedure accounted for 72.02%, while those who underwent multiple procedures accounted for 27.98%. The median preoperative LVEF was 56% (range: 34–67%). According to preoperative NYHA classification, 21.43% were class I, 61.9% were class II, and 16.67% were class III. The high kinesiophobia group showed a lower proportion of college-educated patients (*p* = 0.0286), longer disease duration (*p* < 0.001), higher percentage of patients with monthly income below 5,000 RMB (52.90% vs. 23.33%, *p* = 0.0033), and slightly lower LVEF (*p* = 0.00184) (Table 1). We further analyzed the distribution of chronic comorbidities among patients with different health literacy levels. The results showed that both patients with higher and lower health literacy had hypertension, diabetes, dyslipidemia, and chronic kidney disease. However, the proportion of comorbidities was higher in patients with lower health literacy, although the differences were not statistically significant (Supplementary File 1).

**Table 1:** Differences in baseline characteristics between the high and low kinesiophobia groups.

Variables	All patients (*n* = 168)	Low kinesiophobia (*n* = 30)	High kinesiophobia (*n* = 138)	*p*-value
Age				0.622
≥60	94 (55.95%)	18 (60.00%)	76 (55.07%)	
<60	74 (44.05%)	12 (40.00%)	62 (44.93%)	
Gender				0.880
Male	110 (65.48%)	20 (66.67%)	90 (65.22%)	
Female	58 (34.52%)	10 (33.33%)	48 (34.78%)	
BMI				0.773
≤23.9	80 (47.62%)	15 (50.00%)	65 (47.10%)	
>23.9	88 (52.38%)	15 (50.00%)	73 (52.90%)	
Education levels				0.0286
Junior high school or below	52 (30.95%)	7 (23.33%)	45 (32.61%)	
Senior high school	61 (36.31%)	7 (23.33%)	54 (39.13%)	
College diploma or above	55 (32.74%)	16 (53.33%)	39 (28.26%)	
Marital status				0.283
Married	121 (72.02%)	24 (80.00%)	97 (70.29%)	
Others	47 (27.98%)	6 (20.00%)	41 (29.71%)	
Average monthly household income per capita				0.0033
Less than 5,000 RMB	80 (47.62%)	7 (23.33%)	73 (52.90%)	
5,000 RMB or above	88 (52.38%)	23 (76.67%)	65 (47.10%)	
Payment method				0.236
Medical insurance	153 (91.07%)	29 (96.67%)	124 (89.86%)	
Self-payment	15 (8.93%)	1 (3.33%)	14 (10.14%)	
Living arrangement				0.838
Living with family	70 (41.67%)	13 (43.33%)	57 (41.30%)	
Living alone	98 (58.33%)	17 (56.67%)	81 (58.70%)	
Duration of illness (years)				< 0.001
≥3	91 (54.17%)	5 (16.67%)	86 (62.32%)	
<3	77 (45.83%)	25 (83.33%)	52 (37.68%)	
Number of stents implanted				0.655
Fewer than 2	89 (52.98%)	17 (56.67%)	72 (52.17%)	
2 or more	79 (47.02%)	13 (43.33%)	66 (47.83%)	
Smoking				0.191
Yes	44 (26.19%)	5 (16.67%)	39 (28.26%)	
No	124 (73.81%)	25 (83.33%)	99 (71.74%)	
Alcohol consumption				0.480
Yes	3 (1.79%)	1 (3.33%)	2 (1.45%)	
No	165 (98.21%)	29 (96.67%)	136 (98.55%)	
Angina history				0.988
Yes	73 (43.45%)	13 (43.33%)	60 (43.48%)	
No	95 (56.55%)	17 (56.67%)	78 (56.52%)	
Myocardial infarction history				0.345
Yes	4 (2.38%)	0 (0%)	4 (2.90%)	
No	164 (97.62%)	30 (100%)	134 (97.10%)	
Number of PCI procedures				0.785
Single	121 (72.02%)	21 (70.00%)	100 (72.46%)	
Multiple	47 (27.98%)	9 (30.00%)	38 (27.54%)	
Preoperative left ventricular ejection fraction (LVEF) (%)	56 (34–67)	59 (38–67)	55 (34–67)	0.00184
Preoperative NYHA stage				0.870
NYHA I	36 (21.43%)	6 (20.00%)	30 (21.74%)	
NYHA II	104 (61.9%)	20 (66.67%)	84 (60.87%)	
NYHA III and IV	28 (16.67%)	4 (13.33%)	24 (17.39%)	

### 3.2 The effect of health literacy on kinesiophobia in multivariate logistic regression models adjusted for different factors

**Figure 1:**
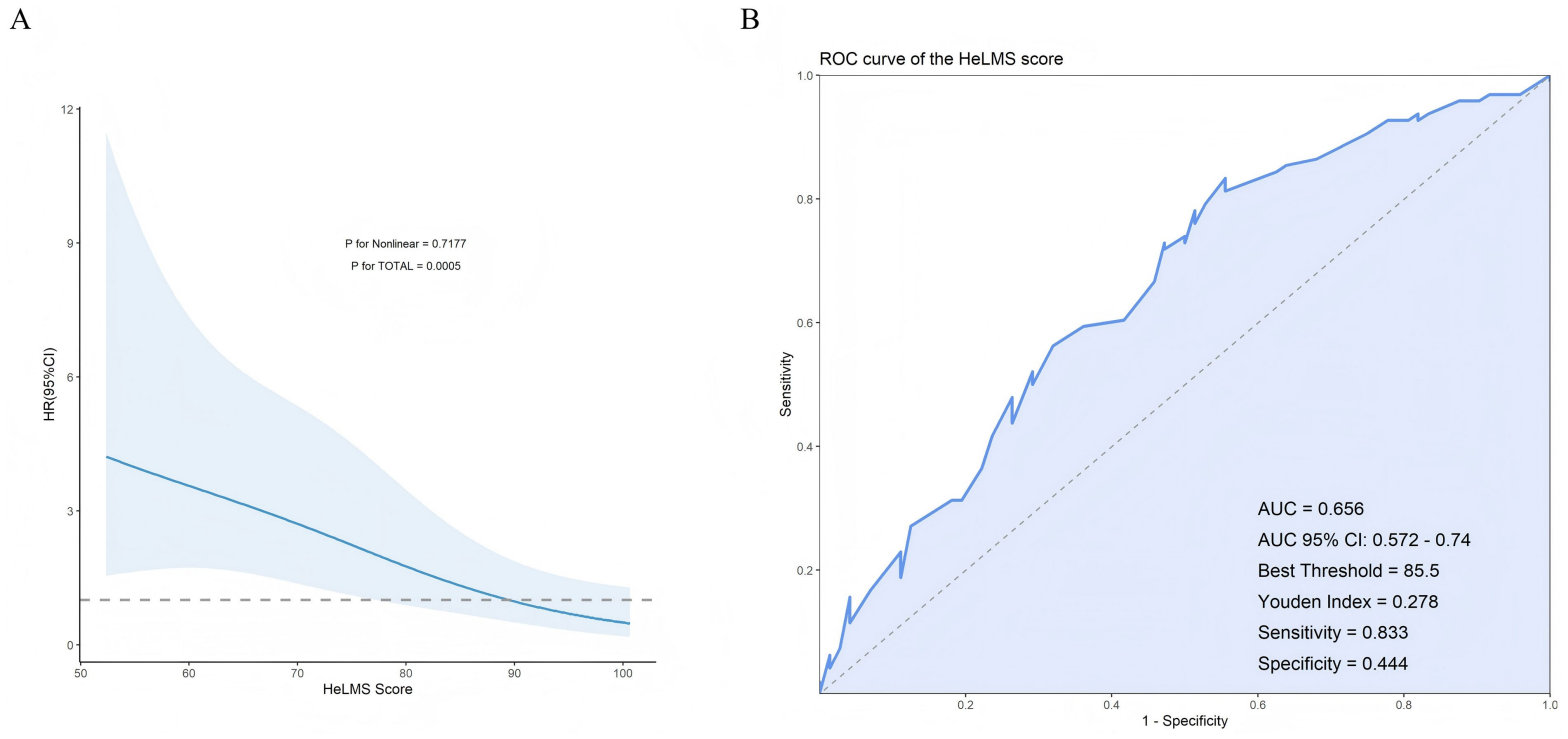
**(A)** Nonlinear analysis of the relationship between HeLMS score and the risk of high kinesiophobia. **(B)** ROC curve of HeLMS for identifying high kinesiophobia.

The results showed that in Model 1 (without adjustment for any covariates), compared with the group with the lowest health literacy (HeLMS Q1, used as the reference), the risk of kinesiophobia gradually decreased in HeLMS Q2, HeLMS Q3, and HeLMS Q4 groups. The differences were statistically significant for HeLMS Q3 (OR = 0.700, 95% CI: 0.588–0.832, *p* < 0.001) and HeLMS Q4 (OR = 0.467, 95% CI: 0.393–0.555, *p* < 0.001). In Model 2 (adjusted for age, gender, and BMI), HeLMS Q3 (OR = 0.693, 95% CI: 0.583–0.825, *p* < 0.001) and HeLMS Q4 (OR = 0.474, 95% CI: 0.397–0.565, *p* < 0.001) remained significantly associated with lower levels of kinesiophobia. In Model 3 (further adjusted for additional confounding factors), HeLMS Q3 (OR = 0.764, 95% CI: 0.650–0.897, *p* = 0.001) and HeLMS Q4 (OR = 0.583, 95% CI: 0.492–0.692, *p* < 0.001) still maintained statistical significance. This indicates that higher health literacy is associated with lower levels of kinesiophobia (Table 2). The complete output table of the model is presented in Supplementary File 2. The calibration curve results showed good agreement between predicted probabilities and observed outcomes. The calibration curves were close to the ideal 45° reference line (Supplementary Figures 2A–C), indicating satisfactory model calibration. To assess the potential impact of other unmeasured confounders, an E-value analysis was performed. The results showed that the E-value was 2.82, indicating that an unmeasured confounder associated with both health literacy and kinesiophobia with a relatively strong association (RR ≥ 2.82) would be required to fully explain the observed association. Therefore, it is unlikely that unmeasured confounders alone could fully account for the observed results. The results of the RCS analysis showed that as the HeLMS score increased, the risk of high kinesiophobia gradually decreased. The overall curve indicated a significant effect of health literacy on kinesiophobia (P for TOTAL <0.05). The relationship between HeLMS score and the risk of high kinesiophobia was not significantly nonlinear (P for Nonlinear = 0.7177), suggesting a linear or near-linear relationship between the two variables (Figure 1A). The ROC curve analysis demonstrated that the HeLMS score had good discriminatory ability for identifying high kinesiophobia, with an area under the curve (AUC) of 0.656 (95% CI: 0.572–0.740). The optimal cutoff value was 85.5, yielding a sensitivity of 0.833 and a specificity of 0.444 (Figure 1B). After performing the multivariable fractional polynomial logistic regression analysis, the regression coefficient for HeLMS (scaled as HeLMS/100) was negative and statistically significant (B = −0.220, *p* < 0.05). A significant negative association between health literacy and kinesiophobia was still observed, suggesting a linear or near-linear relationship (Supplementary File 3).

**Table 2:** Impact of health literacy on kinesiophobia in the multivariable logistic regression model adjusted for different factors.

Model	Term	Estimate	Std error	Statistic	*P* value	OR	CI-lower	CI-upper
Model 1	HeLMSQ1	Ref	Ref	Ref	Ref	Ref	Ref	Ref
	HeLMSQ2	−0.119	0.088	−1.347	0.180	0.888	0.747	1.056
	HeLMSQ3	−0.357	0.088	−4.041	0.000	0.700	0.588	0.832
	HeLMSQ4	−0.762	0.088	−8.620	0.000	0.467	0.393	0.555
Model 2	HeLMSQ1	Ref	Ref	Ref	Ref	Ref	Ref	Ref
	HeLMSQ2	−0.135	0.088	−1.527	0.129	0.874	0.736	1.039
	HeLMSQ3	−0.366	0.088	−4.141	0.000	0.693	0.583	0.825
	HeLMSQ4	−0.747	0.090	−8.315	0.000	0.474	0.397	0.565
Model 3	HeLMSQ1	Ref	Ref	Ref	Ref	Ref	Ref	Ref
	HeLMSQ2	−0.069	0.080	−0.860	0.391	0.933	0.797	1.093
	HeLMSQ3	−0.269	0.082	−3.289	0.001	0.764	0.650	0.897
	HeLMSQ4	−0.539	0.087	−6.197	0.000	0.583	0.492	0.692

### 3.3 Interaction effects of health literacy and other factors on kinesiophobia

**Figure 2:**
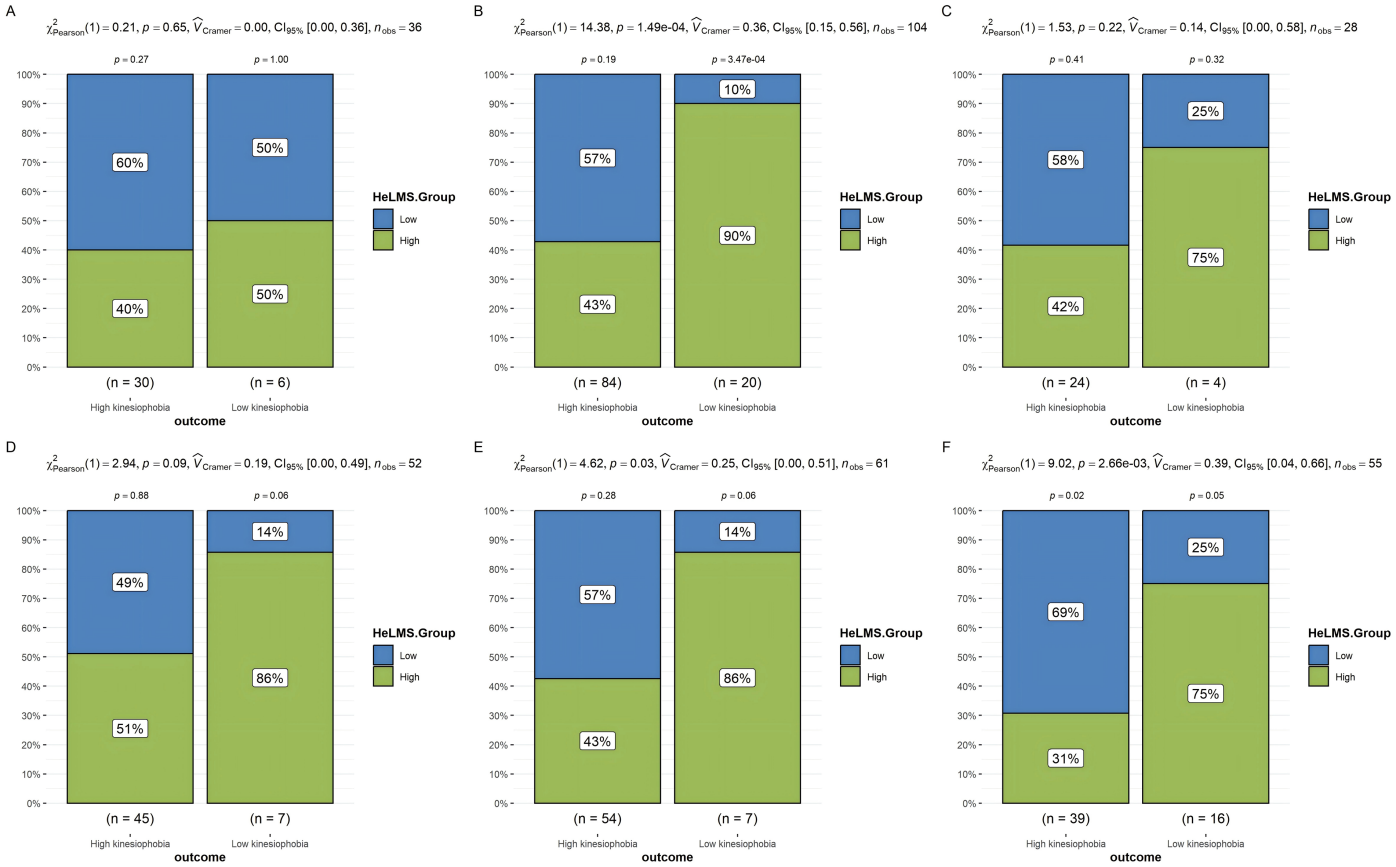
**(A)** Distribution differences of health literacy (HeLMS) groups between high and low kinesiophobia groups in patients with NYHA class I. **(B)** Distribution differences of health literacy (HeLMS) groups between high and low kinesiophobia groups in patients with NYHA class II. **(C)** Distribution differences of health literacy (HeLMS) groups between high and low kinesiophobia groups in patients with NYHA class III. **(D)** Distribution differences of health literacy (HeLMS) groups between high and low kinesiophobia groups in patients with education level of junior high school or below. **(E)** Distribution differences of health literacy (HeLMS) groups between high and low kinesiophobia groups in patients with education level of high school. **(F)** Distribution differences of health literacy (HeLMS) groups between high and low kinesiophobia groups in patients with education level of college or above.

The results showed that the interaction between HeLMS groups and education level significantly affected the risk of high kinesiophobia (OR = 0.426, 95% CI: 0.199–0.913, *p* = 0.028). The stronger the interaction between these two factors, the lower the risk of high kinesiophobia. This indicates that higher education levels may enhance the protective effect of health literacy against high kinesiophobia, meaning that better education potentially strengthens the ability of health literacy to reduce the risk of kinesiophobia. Similarly, the interaction between HeLMS groups and NYHA functional class had a significant impact on the risk of high kinesiophobia (OR = 0.044, 95% CI: 0.005–0.416, *p* = 0.006). The stronger this interaction, the lower the risk of high kinesiophobia. The influence of health literacy on kinesiophobia differs markedly among patients with different cardiac functional statuses. Specifically, health literacy may have a more pronounced protective effect in patients with better cardiac function, significantly lowering the risk of kinesiophobia. Other interactions, such as those with age, disease duration, and number of stents, were not significant (Table 3). We further conducted stratified analyses to validate these findings. Significant differences in HeLMS grouping between high and low kinesiophobia groups were only observed in patients with NYHA class II, further confirming that health literacy significantly reduces kinesiophobia in patients with better cardiac function (Figures 2A–C). As education level increased, the distribution of HeLMS groups between high and low kinesiophobia groups became more significant. Among patients with education levels of high school and above (including college and higher), the proportion of high health literacy was significantly greater in the low kinesiophobia group than in the high kinesiophobia group (Figures 2D–F).

**Table 3:** Interaction effects between health literacy and other factors on kinesiophobia.

Term	Estimate	Std error	Statistic	*P* value	OR	CI-lower	CI-upper
(Intercept)	1.131	0.364	3.111	0.002	3.100	1.520	6.323
HeLMS group	−0.803	0.477	−1.682	0.093	0.448	0.176	1.142
Age group	−0.831	0.468	−1.775	0.076	0.435	0.174	1.090
HeLMS group*Age group	−0.110	0.658	−0.168	0.867	0.896	0.246	3.255

Term	Estimate	Std error	Statistic	*P* value	OR	CI-lower	CI-upper
(Intercept)	0.598	0.375	1.593	0.111	1.818	0.871	3.796
HeLMS group	−0.010	0.523	−0.020	0.984	0.990	0.355	2.758
Education levels	0.056	0.279	0.203	0.839	1.058	0.613	1.827
HeLMS group*Education levels	−0.853	0.389	−2.194	0.028	0.426	0.199	0.913

Term	Estimate	Std error	Statistic	*P* value	OR	CI-lower	CI-upper
(Intercept)	0.961	0.326	2.948	0.003	2.615	1.380	4.956
HeLMS group	−0.799	0.464	−1.722	0.085	0.450	0.181	1.117
Duration group	−0.617	0.455	−1.356	0.175	0.540	0.221	1.316
HeLMS group*Duration group	0.126	0.641	0.196	0.845	1.134	0.323	3.984

Term	Estimate	Std error	Statistic	*P* value	OR	CI-lower	CI-upper
(Intercept)	1.981	0.533	3.714	0.000	7.250	2.549	20.623
HeLMS group	−0.882	0.689	−1.280	0.200	0.414	0.107	1.597
Number of stents group	−1.872	0.598	−3.131	0.002	0.154	0.048	0.497
HeLMS group*Number of stents group	0.051	0.797	0.064	0.949	1.052	0.221	5.019

Term	Estimate	Std error	Statistic	*P* value	OR	CI-lower	CI-upper
(Intercept)	1.386	0.645	2.148	0.032	4.000	1.129	14.175
HeLMS group	−0.182	0.922	−0.198	0.843	0.833	0.137	5.077
Number of PCI group	−0.853	0.690	−1.238	0.216	0.426	0.110	1.645
HeLMS group*Number of PCI group	−0.682	0.985	−0.692	0.489	0.506	0.073	3.486

Term	Estimate	Std error	Statistic	*P* value	OR	CI-lower	CI-upper
(Intercept)	0.758	0.313	2.421	0.015	2.133	1.155	3.939
HeLMS group	−0.145	0.465	−0.311	0.756	0.865	0.348	2.154
LVEF group	−0.208	0.451	−0.461	0.645	0.813	0.336	1.965
HeLMS group*LVEF group	−1.134	0.654	−1.734	0.083	0.322	0.089	1.159

Term	Estimate	Std error	Statistic	*P* value	OR	CI-lower	CI-upper
(Intercept)	1.980	0.709	2.793	0.005	7.243	1.805	29.066
HeLMS group	4.932	2.182	2.260	0.024	138.590	1.923	9985.987
NYHA stage group	−0.846	0.412	−2.052	0.040	0.429	0.191	0.963
HeLMS group*NYHA stage group	−3.113	1.141	−2.728	0.006	0.044	0.005	0.416

### 3.4 Effects of post-PCI pain on health literacy and kinesiophobia

**Figure 3:**
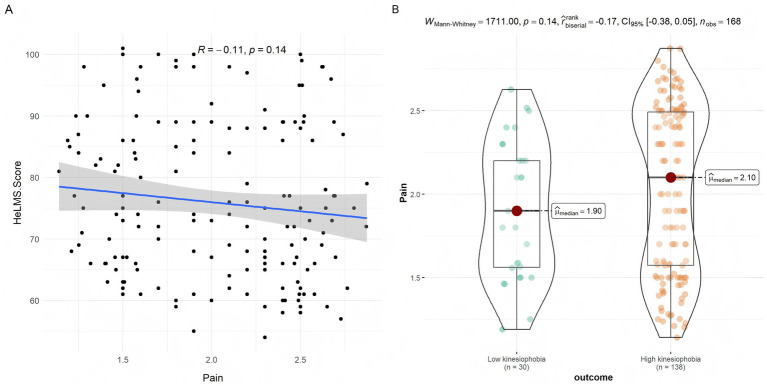
**(A)** Correlation analysis between post-PCI pain levels and HeLMS. **(B)** Comparison of post-PCI pain levels between high and low kinesiophobia groups.

We conducted correlation analyses between postoperative pain levels and health literacy. The results showed that patients’ pain scores were all below VAS = 3, indicating mild postoperative pain. No significant correlation was found between pain intensity and HeLMS scores (r = −0.11, *p* = 0.14) (Figure 3A). We also compared postoperative pain levels between patients with high and low kinesiophobia, and no significant difference was observed (*p* = 0.14) (Figure 3B).

## 4 Discussion

Univariate analysis found that patients in the high kinesiophobia group were significantly older than those in the low kinesiophobia group. This may be because, with increasing age, physiological functions gradually decline (Young, 1997), including decreases in musculoskeletal strength, balance ability (Gusi et al., 2012), and endurance. Elderly patients often lack sufficient exercise experience and training and have inadequate awareness of exercise skills and safety, leading to concerns that physical activity might increase bodily burden or cause adverse outcomes. Older adults tend to overestimate the risks of exercise (e.g., “exercise may trigger a heart attack”) while underestimating its benefits. Such cognitive biases may lead to kinesiophobia. The disease duration was significantly longer in the high kinesiophobia group than in the low group, possibly because chronic illness imposes a greater psychological burden and anxiety, causing patients to worry that exercise might trigger cardiac events or worsen existing cardiovascular conditions, resulting in exercise fear. Additionally, long-term disease status may cause physical function decline and reduced stamina, making patients doubt their exercise capacity and fear they cannot handle the physical demands of exercise, further aggravating kinesiophobia. Compared with the low-kinesiophobia group, the proportion of patients with two or more stents implanted was significantly higher in the high-kinesiophobia group. This may be because multiple stent implantation generally indicates poorer physical condition and more extensive coronary artery disease. Such patients experience greater cardiac burden during daily activities (Bauersachs et al., 2019), leading to angina or dyspnea (Rajkumar et al., 2023), which increases their fear of exercise. Stent implantation may negatively impact patients’ perceptions of their heart health, making them fear that exercise might interfere with surgical site healing or increase complications such as stent displacement or detachment, thereby increasing exercise fear.

The study showed that 96 patients (57.14%) exhibited high kinesiophobia. This may be due to post-PCI patients experiencing discomfort such as pain and fatigue, accompanied by adverse symptoms like chest pain and chest tightness, which increase fear of exercise. Moreover, some post-PCI patients lack confidence in their physical condition and worry that exercise might worsen their illness or cause discomfort, thereby increasing kinesiophobia and health concerns. Health literacy was significantly negatively correlated with kinesiophobia. Patients with higher health literacy better understand disease mechanisms scientifically and accurately, leading to correct perceptions such as knowing that exercise will not cause stent detachment (Hasannejadasl et al., 2022). High health literacy patients are more adept at obtaining and utilizing medical information from reliable sources (e.g., doctors, clinical guidelines) and are less likely to believe in non-professional sources (e.g., internet rumors) (Perrin et al., 2022). They can accurately understand and timely follow prescribed exercise plans, reducing adverse experiences caused by “improper operation.” High literacy patients have higher self-efficacy and trust in their ability to safely follow exercise regimens (e.g., monitoring heart rate to adjust intensity), thus reducing anxiety caused by a sense of “loss of control” (Liu et al., 2023). They also focus more on health behavior management, mastering medication use (e.g., nitrates before exercise) and emergency strategies (e.g., stopping criteria for exercise). Such patients usually have stronger psychological regulation abilities and rationally interpret transient discomfort during exercise without catastrophizing it as a “heart attack.” They hold positive health beliefs, viewing “scientific exercise as improving prognosis” rather than “resting as safer.” Besides, they effectively utilize rehabilitation resources, actively participate in cardiac rehab programs or exercise guidance classes, receive professional support, clearly communicate rehabilitation needs to family members, and negotiate progressive exercise plans (e.g., starting with short-distance walking). Family can assist with recording exercise or accompany exercise, enhancing safety perception and confidence, jointly creating a suitable exercise environment. The ROC analysis indicated that the HeLMS score has potential clinical utility in distinguishing patients with high and low levels of kinesiophobia. The relatively high sensitivity (83.3%) suggests that the scale can effectively identify patients at risk of high kinesiophobia, thereby reducing the likelihood of missing high-risk individuals. However, its specificity was relatively low. Therefore, the HeLMS score may serve as a preliminary screening tool and should be used in conjunction with clinical assessment for comprehensive evaluation.

A strength of this study is the analysis of interaction effects. The results show that as education level increases, the negative association between health literacy and kinesiophobia becomes stronger. The potential mechanism may be that patients with higher education levels have stronger abilities to process and integrate medical information and more easily translate health knowledge into practice, thereby reducing irrational fears of exercise. They tend to consult official guidelines to correct incorrect rehabilitation concepts. The interaction between health literacy and NYHA functional classification suggested that patients with NYHA class II or above, who often experience symptoms during daily activities (e.g., dyspnea, fatigue), may find it difficult for health literacy alone to overcome the fear caused by physical discomfort (Gilbert and Xu, 2017). In contrast, the effect of health knowledge is more evident in class I patients with mild symptoms. Patients with poorer cardiac function may experience more exercise restrictions and feelings of frustration, leading to a vicious cycle of “learned helplessness” where they feel “no matter how hard I try, there is no improvement.” Even though they know exercise benefits recovery, the disconnect between physical symptoms and cognition leads to failure of health literacy to moderate fear. These findings remind us that in clinical practice, patients with higher education and better cardiac function benefit more from health literacy, whereas those with lower education and worse cardiac function may require integrated psychological support or personalized rehabilitation plans. For example, simpler health education tools combined with symptom management (e.g., optimizing diuretic use) and development of stratified, differentiated health education content tailored to cardiac function levels. It should be noted that, in the interaction analysis of this study, some continuous variables were categorized, which may result in partial loss of information and reduced statistical efficiency. Therefore, the interaction results should be interpreted with caution and can be regarded as exploratory analyses, aiming to preliminarily explore potential interactions between variables.

Our findings indicate that postoperative pain after PCI was generally low and not significantly associated with either health literacy or kinesiophobia, which is unsurprising. This may be due to the minimally invasive nature of PCI, the small operative trauma, and the short procedure duration. Even in patients undergoing multi-stent procedures, postoperative pain generally remained mild, consistent with previous studies (Brogiene et al., 2022). The relatively low pain levels have limited impact on health literacy and kinesiophobia because the assessment of these factors mainly relies on patients’ cognitive ability and knowledge, and mild short-term physical discomfort does not affect comprehension or questionnaire responses.

This study also has certain limitations. First, it is a cross-sectional study and may have selection bias. Second, it is a single-center study with a relatively small sample size. Third, both health literacy and kinesiophobia were assessed by questionnaires, which involve subjective cognitive differences and recall bias. In addition, patients’ postoperative health decisions may be affected by external sources of information such as social media, the internet, and print media, which could potentially confound the results. This study did not fully adjust for potential confounding factors, including depression, anxiety, pain catastrophizing, illness perceptions, social support, participation in cardiac rehabilitation, and physician-imposed activity restrictions, which may have influenced the study results. Future studies should consider using multidimensional assessment tools, conducting prospective, large-scale, multicenter randomized controlled trials, and incorporating analyses of the influence of external information sources to further validate and expand the findings of this study. For the stratified analysis, the relatively small number of patients in NYHA class III and those with a college degree or above may lead to limited statistical power, potentially affecting the stability and reliability of the results.

## 5 Conclusion

This study indicates that factors such as age, education level, and disease duration are significantly associated with kinesiophobia after PCI. Health literacy shows a significant linear or near-linear negative correlation with kinesiophobia. In patients with higher education levels and better cardiac function, this association is more pronounced.

## Data Availability

The original contributions presented in the study are included in the article/Supplementary material, further inquiries can be directed to the corresponding author.

## Supplementary material

SUPPLEMENTARY FIGURE 1 Patient enrollment flowchart.

SUPPLEMENTARY FIGURE 2 **(A)** Calibration curve of model 1; **(B)** Calibration curve of model 2; **(C)** Calibration curve of model 3.
