# Supplementary figures and images for: Association between health literacy and kinesiophobia in patients after percutaneous coronary intervention
Source: Front Psychol. 2026 Jul 2;17:1689455. doi: 10.3389/fpsyg.2026.1689455 (PMC13373948; doi:10.3389/fpsyg.2026.1689455)

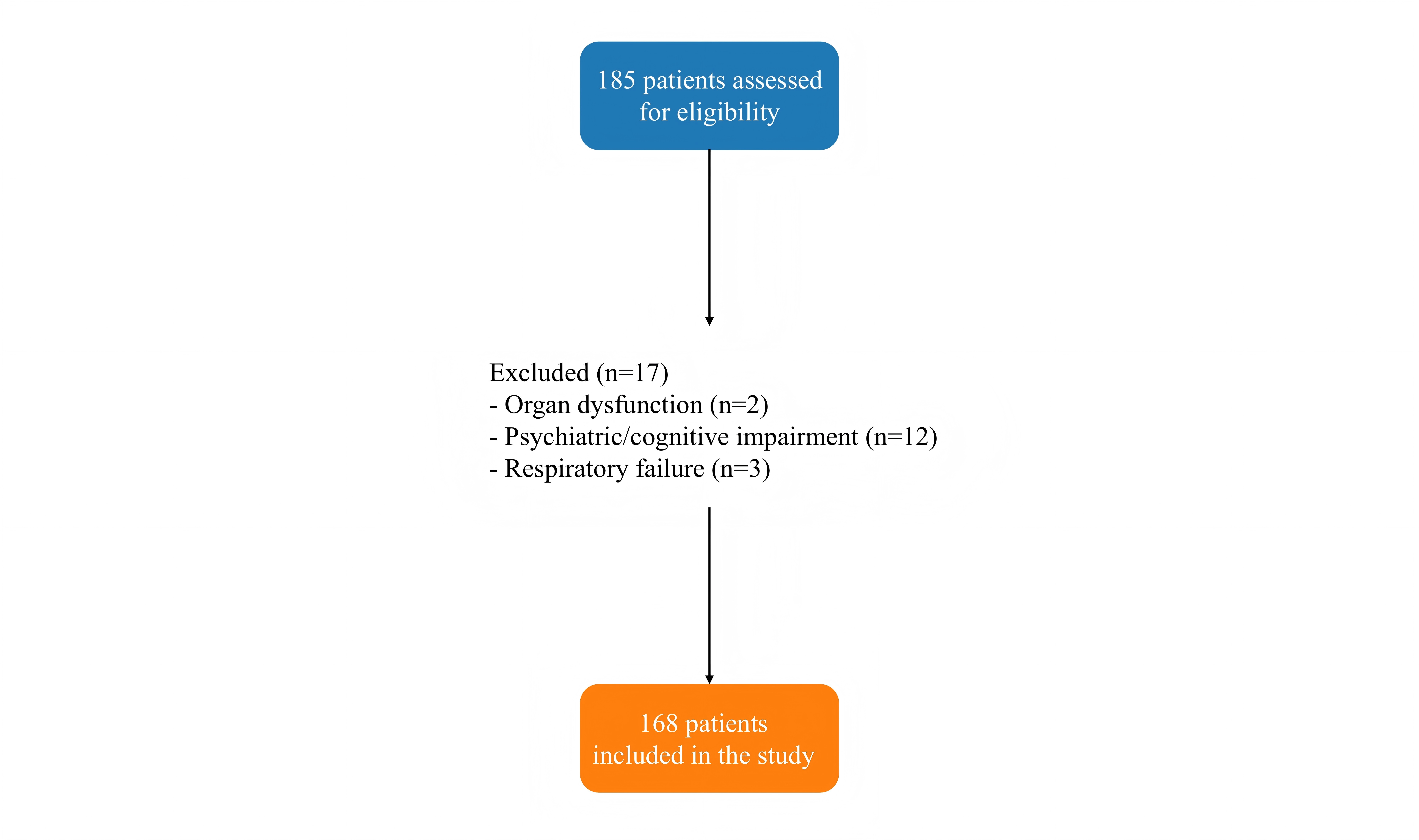

Supplement: SUPPLEMENTARY FIGURE 1 — Patient enrollment flowchart. [file Image_1.JPEG]

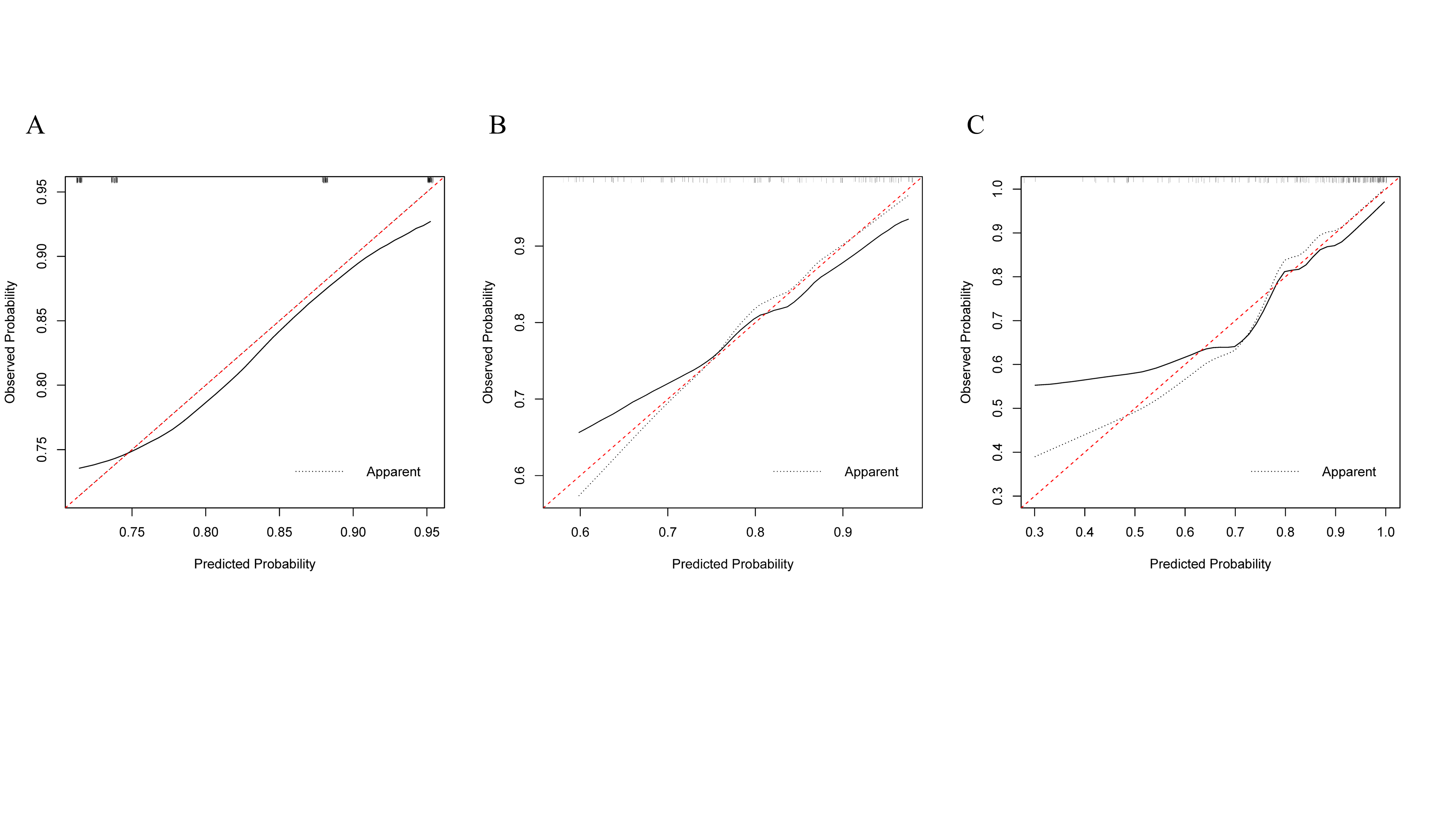

Supplement: SUPPLEMENTARY FIGURE 2 — (A) Calibration curve of model 1; (B) Calibration curve of model 2; (C) Calibration curve of model 3. [file Image_2.TIF]
