# Supplementary material for: Association between health literacy and kinesiophobia in patients after percutaneous coronary intervention
Source: Front Psychol. 2026 Jul 2;17:1689455. doi: 10.3389/fpsyg.2026.1689455 (PMC13373948; doi:10.3389/fpsyg.2026.1689455)
Supplement: Supplementary file 3 [file Supplementary_file_1.docx]

| **Supplementary Table. 1 Distribution of Chronic Comorbidities Across Different Health Literacy Groups** | | | | | |
| --- | --- | --- | --- | --- | --- |
| **Comorbidity** | **HeLMSQ1** | **HeLMSQ2** | **HeLMSQ3** | **HeLMSQ4** | **P-value** |
| **Hypertension** | 33 (78.57%) | 26 (61.9%) | 27 (64.29%) | 27 (64.29%) | 0.344 |
| **Diabetes Mellitus** | 16 (38.1%) | 11 (26.19%) | 12 (28.57%) | 6 (14.29%) | 0.104 |
| **Dyslipidemia** | 23 (54.76%) | 21 (50%) | 14 (33.33%) | 14 (33.33%) | 0.093 |
| **Chronic Kidney Disease (CKD)** | 7 (16.67%) | 3 (7.14%) | 4 (9.52%) | 1 (2.38%) | 0.139 |
