# Supplementary material for: Association between health literacy and kinesiophobia in patients after percutaneous coronary intervention
Source: Front Psychol. 2026 Jul 2;17:1689455. doi: 10.3389/fpsyg.2026.1689455 (PMC13373948; doi:10.3389/fpsyg.2026.1689455)
Supplement: Supplementary file 4 [file Supplementary_file_2.docx]

| **Supplementary Table. 2 Full results of Model 1, Model 2, and Model 3** | | | | | | | |
| --- | --- | --- | --- | --- | --- | --- | --- |
| **Parameter** | **Estimate** | **SE** | **Statistic** | **P value** | **OR** | **CI-lower** | **CI-upper** |
| **Model 1** |  |  |  |  |  |  |  |
| **(Intercept)** | 0.881 | 0.062 | 14.096 | 0.000 | 2.413 | 2.135 | 2.728 |
| **HeLMSQ2** | -0.119 | 0.088 | -1.347 | 0.180 | 0.888 | 0.747 | 1.056 |
| **HeLMSQ3** | -0.357 | 0.088 | -4.041 | 0.000 | 0.700 | 0.588 | 0.832 |
| **HeLMSQ4** | -0.762 | 0.088 | -8.620 | 0.000 | 0.467 | 0.393 | 0.555 |
| **Model 2** |  |  |  |  |  |  |  |
| **(Intercept)** | 0.120 | 0.381 | 0.316 | 0.753 | 1.128 | 0.535 | 2.378 |
| **HeLMSQ2** | -0.135 | 0.088 | -1.527 | 0.129 | 0.874 | 0.736 | 1.039 |
| **HeLMSQ3** | -0.366 | 0.088 | -4.141 | 0.000 | 0.693 | 0.583 | 0.825 |
| **HeLMSQ4** | -0.747 | 0.090 | -8.315 | 0.000 | 0.474 | 0.397 | 0.565 |
| **Age** | 0.007 | 0.003 | 2.134 | 0.034 | 1.007 | 1.001 | 1.014 |
| **Gender** | -0.061 | 0.066 | -0.927 | 0.356 | 0.941 | 0.827 | 1.071 |
| **BMI** | 0.015 | 0.013 | 1.188 | 0.237 | 1.015 | 0.990 | 1.040 |
| **Model 3** |  |  |  |  |  |  |  |
| **(Intercept)** | 0.944 | 0.731 | 1.293 | 0.198 | 2.571 | 0.614 | 10.764 |
| **HeLMSQ2** | -0.069 | 0.080 | -0.860 | 0.391 | 0.933 | 0.797 | 1.093 |
| **HeLMSQ3** | -0.269 | 0.082 | -3.289 | 0.001 | 0.764 | 0.650 | 0.897 |
| **HeLMSQ4** | -0.539 | 0.087 | -6.197 | 0.000 | 0.583 | 0.492 | 0.692 |
| **Age** | 0.006 | 0.003 | 1.962 | 0.052 | 1.006 | 1.000 | 1.012 |
| **Gender** | -0.089 | 0.061 | -1.467 | 0.144 | 0.914 | 0.811 | 1.030 |
| **BMI** | 0.007 | 0.011 | 0.620 | 0.536 | 1.007 | 0.985 | 1.030 |
| **Education levels** | -0.028 | 0.036 | -0.784 | 0.434 | 0.972 | 0.907 | 1.043 |
| **Duration** | 0.026 | 0.015 | 1.703 | 0.091 | 1.026 | 0.996 | 1.057 |
| **Number of stents** | 0.155 | 0.063 | 2.473 | 0.015 | 1.167 | 1.033 | 1.320 |
| **Myocardial infarction history** | 0.124 | 0.058 | 2.147 | 0.033 | 1.132 | 1.011 | 1.269 |
| **Number of PCI** | 0.055 | 0.078 | 0.706 | 0.482 | 1.057 | 0.907 | 1.232 |
| **LVEF** | -0.018 | 0.012 | -1.480 | 0.141 | 0.982 | 0.959 | 1.006 |
| **NYHA stage** | 0.205 | 0.049 | 4.177 | 0.000 | 1.228 | 1.115 | 1.352 |
