# Supplementary material for: Association between health literacy and kinesiophobia in patients after percutaneous coronary intervention
Source: Front Psychol. 2026 Jul 2;17:1689455. doi: 10.3389/fpsyg.2026.1689455 (PMC13373948; doi:10.3389/fpsyg.2026.1689455)
Supplement: Supplementary file 5 [file Supplementary_file_3.docx]

| **Supplementary Table. 3 Multivariable fractional polynomial logistic regression analysis of factors associated with kinesiophobia** | | | | | | | |
| --- | --- | --- | --- | --- | --- | --- | --- |
| **Variable** | **Estimate** | **SE** | **Statistic** | **P value** | **OR** | **CI-lower** | **CI-upper** |
| **NYHA stage** | 1.220 | 0.427 | 2.859 | 0.004 | 3.389 | 1.468 | 7.823 |
| **Number of stents** | 1.395 | 0.616 | 2.266 | 0.023 | 4.036 | 1.207 | 13.491 |
| **Education levels** | -0.719 | 0.326 | -2.206 | 0.027 | 0.487 | 0.257 | 0.923 |
| **LVEF** | -0.547 | 1.922 | -0.285 | 0.776 | 0.579 | 0.013 | 25.005 |
| **Myocardial infarction history** | 0.968 | 0.526 | 1.839 | 0.066 | 2.633 | 0.938 | 7.386 |
| **Age** | -0.036 | 0.026 | -1.376 | 0.169 | 0.964 | 0.916 | 1.016 |
| **Number of PCI** | 0.598 | 0.711 | 0.841 | 0.400 | 1.818 | 0.451 | 7.325 |
| **Gender** | -0.432 | 0.525 | -0.823 | 0.410 | 0.649 | 0.232 | 1.815 |
| **Duration** | 0.076 | 0.130 | 0.583 | 0.560 | 1.079 | 0.836 | 1.391 |
| **I((HeLMS Score/100)^1)** | -0.220 | 0.103 | -2.135 | 0.033 | 0.803 | 0.656 | 0.982 |
| **BMI** | -0.016 | 0.090 | -0.172 | 0.863 | 0.985 | 0.825 | 1.176 |
